# The Incorporated Drug Affects the Properties of Hydrophilic Nanofibers

**DOI:** 10.3390/nano14110949

**Published:** 2024-05-28

**Authors:** Črt Dragar, Robert Roškar, Petra Kocbek

**Affiliations:** 1 Department of Pharmaceutical Technology, Faculty of Pharmacy, University of Ljubljana, SI-1000 Ljubljana, Slovenia; crt.dragar@ffa.uni-lj.si; 2 Department of Biopharmaceutics and Pharmacokinetics, Faculty of Pharmacy, University of Ljubljana, SI-1000 Ljubljana, Slovenia; robert.roskar@ffa.uni-lj.si

**Keywords:** carvedilol, drug loading, electrospinning, ibuprofen, metformin, nanodelivery systems, nanofibers, paracetamol, poloxamer 188, polyethylene oxide

## Abstract

Hydrophilic nanofibers offer promising potential for the delivery of drugs with diverse characteristics. Yet, the effects of different drugs incorporated into these nanofibers on their properties remain poorly understood. In this study, we systematically explored how model drugs, namely ibuprofen, carvedilol, paracetamol, and metformin (hydrochloride), affect hydrophilic nanofibers composed of polyethylene oxide and poloxamer 188 in a 1:1 weight ratio. Our findings reveal that the drug affects the conductivity and viscosity of the polymer solution for electrospinning, leading to distinct changes in the morphology of electrospun products. Specifically, drugs with low solubility in ethanol, the chosen solvent for polymer solution preparation, led to the formation of continuous nanofibers with uniform diameters. Additionally, the lower solubility of metformin in ethanol resulted in particle appearance on the nanofiber surface. Furthermore, the incorporation of more hydrophilic drugs increased the surface hydrophilicity of nanofiber mats. However, variations in the physicochemical properties of the drugs did not affect the drug loading and drug entrapment efficiency. Our research also shows that drug properties do not notably affect the immediate release of drugs from nanofibers, highlighting the dominant role of the hydrophilic polymers used. This study emphasizes the importance of considering specific drug properties, such as solubility, hydrophilicity, and compatibility with the solvent used for electrospinning, when designing hydrophilic nanofibers for drug delivery. Such considerations are crucial for optimizing the properties of the drug delivery system, which is essential for achieving therapeutic efficacy and safety.

## 1. Introduction

In the last three decades, nanofibers have gained tremendous attention in various applications due to their rapid development and the deepened understanding of their preparation [1,2]. These nanostructures, defined as ultrafine fibers with nanoscale diameters and theoretically unlimited length, show unique properties, such as large surface area and the ability to form a highly porous three-dimensional network with nanosized intrafibrillar pores [3,4]. The high surface area-to-volume ratio results in distinct properties of nanofibers compared to their bulk counterparts, showing the potential to revolutionize numerous fields, including filtration, separation, catalysis, electronics, textiles, and biomedicine [2,5].

Nanofibers can be prepared by various methods, among which electrospinning is the most commonly used due to its simplicity [5]. It allows the preparation of nanofibers with defined morphology and nano- or micrometer diameter in a single step, is suitable for continuous production at an industrial scale, and is considered the most economical method for the preparation of nanofibers [2,6,7]. During electrospinning, a viscoelastic jet of a conductive solution is elongated and bent in the electric field established between a grounded collector and a metal needle connected to a high-voltage supply [1,8,9]. This process results in the reduction of jet diameter from several hundred micrometers to nanometer size. The simultaneous rapid evaporation of the solvent leads to the formation of solid nanofibers deposited on the grounded collector [6,9]. The electrospinning process and nanofiber properties are influenced by various factors such as solution properties, process parameters, and ambient conditions, which have been thoroughly described in the literature [1,5,10,11]. The complex interplay of these factors makes it difficult to accurately predict the properties of the produced nanofibers [9]. The most extensively investigated physicochemical properties of nanofibers include their morphology [12], diameter [13], inter- and intrafibrilar porosity [14], crystallinity [15], mechanical properties (e.g., strength and flexibility) [16,17], swelling of nanofibers [17], intermolecular interactions [18], and surface properties [19]. Moreover, the biological properties of nanofibers, such as biocompatibility, immunomodulatory effects, cell mechanosensing, and effect on cells or tissues, are also considered important aspects of nanofiber characterization [17,20]. However, when nanofibers are designed as drug delivery systems, their crucial characteristics include drug loading, drug entrapment efficiency, drug release kinetics, and their performance in vivo [6].

To date, nanofibers have been prepared from more than 100 various polymers, which enable the formation of nanofibers with different properties [21]. Thus, nanofibers can be designed for different routes of administration and to deliver drugs with diverse characteristics (including hydrophilic and hydrophobic drugs, small molecules, and biomacromolecules) and enable various drug-release kinetics [6,8]. Many types of drugs have already been incorporated into different polymer nanofibers, including antimicrobials (e.g., metronidazole [15], ciprofloxacin [22,23], fluconazole [24], moxifloxacin [25], nisin [26], ofloxacin [27], vancomycin [28,29], voriconazole [30], tetracycline [31]), antioxidants (e.g., curcumin [32], resveratrol [33]), anti-inflammatory drugs (e.g., diclofenac [34], naproxen [35], ketoprofen [36], ibuprofen [37,38], indomethacin [39], celecoxib [40], meloxicam [41], dexamethasone [42,43], prednisone [44]), peptide-based drugs (e.g., epidermal growth factor [45,46], insulin [47]), and others (e.g., paracetamol [48,49], cyclosporine A [50], tenofovir [51], carvedilol [52,53], lovastatin [54], simvastatin [55], lidocaine [56], donepezil [57], metformin [58,59], bevacizumab [60]). The drug loading efficiency varies depending on the polymer type, the drug properties, and the method of drug loading. This results in nanofibers with from as little as a few percent [34,61] up to 60% (*w*/*w*) of drug-loaded [62]. The majority of the nanofibers is usually composed of polymers, which thus play an important role in defining the desired properties of the nanofibers [21]. However, as the nanofiber formulation is usually developed for a specific drug in combination with the specific polymers, the substitution of the drug in the formulation can have a significant impact not only on the drug loading [6] but also on other properties of nanofibers.

Thus, the aim of this research was to systematically investigate the influence of the drug on the physicochemical properties of the hydrophilic nanofibers, composed of polyethylene oxide (PEO) and poloxamer 188 (P188). Ibuprofen, carvedilol, paracetamol, and metformin hydrochloride were thus selected as model drugs with different properties.

## 2. Materials and Methods

### 2.1. Materials

All materials used were of reagent grade and from commercial sources. Ibuprofen was from Fagron Hellas (Trikala, Greece), carvedilol was a gift from Krka d.d. (Novo mesto, Slovenia), paracetamol was a gift from Lek d.d. (Ljubljana, Slovenia), and metformin hydrochloride was a gift from Zentiva k.s. (Prague, Czech Republic). Polyethylene oxide (PEO; Mw, 400,000 g/mol) was from Sigma-Aldrich, Co. (St. Louis, MO, USA) and poloxamer 188 (P188; Lutrol^®^ F68) from BASF (Ludwigshafen, Germany). Hydrochloric acid (37%, *w*/*w*; HCl), orthophosphoric acid (85%, *w*/*w*; H_3_PO_4_), sodium hydroxide (NaOH), potassium dihydrogen phosphate (KH_2_PO_4_), formic acid (98–100%, *w*/*w*, HCOOH), and polysorbate 80 (Tween^®^ 80) were from Merck KGaA (Darmstadt, Germany). Acetonitrile was from J.T. Baker (Gliwice, Poland), and ethanol (96%, *v*/*v*) was from Pharmachem Sušnik Jožef (Ljubljana, Slovenia). The water used was purified by reverse osmosis and Milli-Q water was obtained by the Millipore Milli-Q lab water system.

### 2.2. Preparation and Evaluation of Polymer Solutions

To prepare the polymer solutions for electrospinning (Table 1), the polymers, namely PEO and P188 in a weight ratio of 1:1, were dissolved in ethanol at 50 °C by moderate magnetic stirring. The obtained polymer solution was cooled to room temperature before adding the selected drug (Table 1). The drug was dissolved in the polymer solution at room temperature using moderate magnetic stirring. The polymer solution without the drug (formulation 0) was prepared using the same procedure without the addition of the drug.

#### 2.2.1. Rheology Measurements

The rheological properties of the polymer solutions were analyzed using a Physica MCR 301 rheometer (Anton Paar; Graz, Austria) equipped with a cone-plate measuring system CP50-2 (cone diameter, 49.961 mm; cone angle, 2.001°; sample thickness, 0.209 mm). The rotational test was conducted at a controlled shear rate from 1 s^−1^ to 100 s^−1^ at 25 ± 0.1 °C to determine the viscosity of the polymer solutions. The elastic and plastic modulus were evaluated through the oscillatory frequency sweep test, which was conducted at 25 ± 0.1 °C, with a standard strain amplitude of 1%, and varying the angular frequency from 0.1 rad/s to 100 rad/s.

#### 2.2.2. Electrical Conductivity Measurements

The electrical conductivity of the polymer solutions was determined at 25 °C using an MC226 Conductivity Meter with InLab^®^ 741 electrode (Mettler-Toledo; Greifensee, Switzerland). The measurements were performed in triplicates, and the results are given as the average conductivity with corresponding standard deviation.

### 2.3. Electrospinning of Nanofibers

The polymer solutions were electrospun using Spinbox Systems^®^ electrospinning device (Bioinicia; Valencia, Spain) in a horizontal configuration as follows. An aliquot (~5 mL) of the freshly prepared polymer solution was transferred into a 5 mL plastic syringe (Chirana; Stará Turá, Slovakia), which was then placed into a syringe pump of the electrospinning device. The syringe was connected to a metal needle (outer diameter, 0.7 mm; Bioinicia; Valencia, Spain) with a plastic tube (outer diameter, 1.3 mm). The grounded collector was positioned 15 cm away from the tip of the metal needle. The polymer solution was electrospun at a flow rate of 1.77 mL/h and electrical voltage of 15 kV for ~2 h at room temperature and relative humidity ≤ 45%. The obtained nanofibers were stored in a desiccator for at least 12 h before further use.

To prepare polymer films with the same composition as the electrospun nanofibers and similar thickness to the nanofiber mats, ~5 g of the prepared polymer solution (Table 1) was transferred into a Petri dish (inner diameter, 70 mm) and dried at 50 °C for ~1 h. The obtained polymer films were stored in a desiccator for at least 12 h before further use.

To prepare a physical mixture of drug and polymers with the same composition as the electrospun nanofibers, 150 mg of PEO, 150 mg of P188, and 75 mg of the selected drug (except for formulation 0) were weighed and mixed thoroughly in a mortar.

### 2.4. Evaluation of Nanofibers

#### 2.4.1. Scanning Electron Microscopy

The morphology of nanofibers and polymer films was evaluated by scanning electron microscopy (SEM; Supra35 VP, Carl Zeiss; Oberkochen, Germany). The samples were attached to metal studs with double-sided conductive tape (diameter, 12 mm; Oxford Instruments; Oxon, UK), and imaging was performed at an accelerating voltage of 1 kV with the secondary electron detector. At least 100 measurements of nanofiber diameter were performed on representative SEM images using the ImageJ (v1.54d) software (National Institutes of Health; Bethesda, MD, USA), and the average nanofiber diameter, along with the corresponding standard deviation, was calculated.

#### 2.4.2. Fourier-Transform Infrared Analysis

To assess potential chemical interactions between the components, nanofibers, polymer films, and physical mixtures were analyzed using the Fourier-transform infrared (FT-IR) spectrometer Nexus with an attenuated total reflectance accessory (Thermo Nicolet, Madison, WI, USA). The spectra were recorded in the range of 600–3900 cm^−1^ with 64 scans at a resolution of 2 cm^−1^. Additionally, the FT-IR spectra of the individual powdered components were recorded.

#### 2.4.3. Contact Angle Measurements

The surface properties of nanofibers were evaluated by measuring the contact angle between a water droplet and the surface of a nanofiber mat. A piece of nanofiber mat (~1 cm^2^) was attached to a glass slide with double-sided tape Patafix (UHU GmbH & Co, Bühl, Germany), and a 5 µL droplet of water was placed on the surface of the nanofiber mat. The contact angle was determined 0.96 s after the first contact between the drop and the nanofiber mat using a contact angle meter DSA 100 (Krüss; Hamburg, Germany). The measurement was repeated 20 times for each formulation. The results are given as an average contact angle with the corresponding standard deviation.

#### 2.4.4. Thermogravimetric Analysis

The residual moisture content in the nanofibers and polymer films was evaluated by thermogravimetric analysis (TGA) using the thermogravimeter TGA/DSC 1 STARe System (Mettler-Toledo; Greifensee, Switzerland). A sample (~5 mg) was weighed in an aluminum oxide crucible (70 µL) and placed in the cell of a thermogravimeter with an inert atmosphere (nitrogen flow 50 mL/min). The sample was heated at a rate of 30 °C/min from 30 °C to 95 °C, where it was held isothermically for 30 min before being heated at a rate of 20 °C/min up to 250 °C. The residual moisture content was calculated using Equation (1):

(1) $$w_{MOISTURE}=\frac{m_{LOSS\left(30–110\right)}}{m_{0}}\times 100\%$$

where m_0_ is the initial mass of the sample, and m_LOSS (30–110)_ is the mass loss over the temperature range from 30 °C to 110 °C estimated by TGA. The measurements were performed in triplicates, and the results are given as the average residual moisture content with the corresponding standard deviation.

To investigate the moisture sorption ability of nanofibers and polymer films, samples weighing ~5 mg were placed in a chamber maintained at a constant relative humidity of 46% at room temperature for 24 h. The samples were then analyzed by TGA following the previously described procedure. Each experiment was performed in triplicate, and the results are presented as the average moisture content along with the corresponding standard deviation.

#### 2.4.5. Evaluation of Nanofiber Dispersibility

The dispersibility of nanofibers in phosphate buffer (pH 7.4) containing 0.1% (*w*/*v*) Tween^®^ 80 was evaluated by adding ~10 mg of nanofibers to a 20 mL glass vial. Subsequently, 10 mL of the aforementioned phosphate buffer (pH 7.4) with 0.1% (*w*/*v*) Tween^®^ 80 was added to the vial, followed by vortexing until no visible nanofiber residuals or aggregates were observed. The time required for the complete disintegration of the nanofibers was recorded. Each experiment was performed in triplicate, and the results are presented as the average time for nanofiber disintegration along with the corresponding standard deviation.

### 2.5. Determination of Drug Loading in Nanofibers

The drug loading in nanofibers was determined by dissolving ~10 mg of precisely weighed nanofibers in 5 mL of ethanol in a 20 mL measuring flask. The flask was then sonicated (Sonis 4, Iskra PIO, Šentjernej, Slovenia) for 15 min to ensure a complete nanofiber disintegration and drug dissolution. The resulting solution was cooled to room temperature, diluted with ethanol to 20 mL, and stirred moderately with a magnetic stirrer for 30 min. The solution was then filtered through a 0.20 µm hydrophilic cellulose filter (Minisart^®^ RC, Sartorius, Göttingen, Germany) and analyzed for drug content by high-performance liquid chromatography (HPLC) as described in Section 2.7. The drug content (w_DRUG_) was calculated using Equation (2):

(2) $$w_{DRUG}=\frac{c_{HPLC}\;\times 20\;mL}{m_{NF}}\times 100\%\;(w/w)$$

where c_HPLC_ is the drug concentration determined by HPLC analysis, and m_NF_ is the precise mass of nanofibers used in the determination of drug loading.

In the case of formulation CAR, the obtained ethanol solution was diluted 20 times with phosphate buffer (pH 7.4) with 0.1% (*w*/*v*) Tween^®^ 80 before filtering through a 0.20 µm hydrophilic cellulose filter (Minisart^®^ RC, Sartorius, Göttingen, Germany) and HPLC analysis. The determination of drug loading was conducted in triplicates using nanofiber samples collected from various locations on the grounded collector. The results are presented as the average drug loading alongside the corresponding standard deviation.

Based on the determined drug loading, the drug entrapment efficiency (EE) was calculated using Equation (3):

(3) $$EE=\frac{w_{DRUG}}{20\%\;(w/w)}\times 100\%$$

where w_DRUG_ is the drug loading determined as described above, while 20% (*w*/*w*) is the theoretical drug loading based on the composition of polymer solutions (Table 1).

### 2.6. Evaluation of Drug Release In Vitro

To evaluate the in vitro release of the drug from the nanofibers and polymer films, ~10 mg of the sample (~5 mg for formulation CAR) was accurately weighed and gently rolled onto a plastic support and placed in a 20 mL glass vial. Next, 10 mL (75 mL for formulation CAR) of phosphate buffer (pH 7.4) with 0.1% (*w*/*v*) Tween^®^ 80 was added, and the vial was incubated on a shaker (220 rpm, 37 °C, 24 h). At predetermined time points, a 750 µL aliquot was withdrawn and centrifuged at 19,400× *g* for 4 min at 15 °C (Fresco 21, Thermo Fischer Scientific, Osterode am Harz, Germany). The drug content in the supernatant was determined by HPLC analysis as described in Section 2.7. Dissolution of the drug in the form of a physical mixture was evaluated by the same procedure using ~10 mg of precisely weighed physical mixture. All experiments were performed in triplicates under sink conditions. Results are given as the mean percentage of drug released with the corresponding standard deviation for each time point.

### 2.7. HPLC Analysis

The drug content in the samples was determined by HPLC analysis (Agilent 1100, Hewlett Packard, Walbronn, Germany) with a diode array module detector. For ibuprofen analysis, a C18 chromatographic column Luna^®^ (5 µm, 100 Å, 150 mm × 4.6 mm; Phenomenex, Torrance, CA, USA) was used; for carvedilol analysis, a C8 chromatographic column BetaBasic^®^ (3 µm, 150 Å, 150 mm × 4.6 mm; Thermo Fisher Scientific, Waltham, MA, USA) was used; for paracetamol analysis, a C18 chromatographic column Kinetex^®^ (2.6 µm, 150 Å, 50 mm × 4.6 mm; Phenomenex, Torrance, CA, USA) was used; and for metformin hydrochloride analysis, a reverse phase chromatographic column Synergi^TM^ Hydro-RP (4 µm, 80 Å, 150 mm × 4.6 mm; Phenomenex, Torrance, CA, USA) was used.

The mobile phase for ibuprofen analysis consisted of 0.1% (*w*/*w*) H_3_PO_4_ and acetonitrile in a volume ratio of 35:65; for carvedilol analysis, 0.02 M KH_2_PO_4_ and acetonitrile in a volume ratio of 65:35; for paracetamol analysis, 0.01% (*w*/*w*) HCOOH and acetonitrile in a volume ratio of 92:8; and for metformin hydrochloride analysis, 0.01% (*w*/*w*) HCOOH and acetonitrile in a volume ratio of 30:70. The parameters of the HPLC analyses are summarized in Table 2.

The drug content was determined based on the calibration curve, prepared with standard solutions of drugs either in phosphate buffer (pH 7.4) with 0.1% (*w*/*v*) Tween^®^ 80 or in ethanol.

The concentration range for ibuprofen, paracetamol, and metformin hydrochloride determination was from 12.50 µg/mL to 200.00 µg/mL. For carvedilol, the calibration curve was prepared in phosphate buffer (pH 7.4) with 0.1% (*w*/*v*) Tween^®^ 80, in a concentration range from 1.25 µg/mL to 11.25 µg/mL.

### 2.8. Statistical Analysis

The data are given as the average ± standard deviation. To compare the samples statistically, a one-way analysis of variance (ANOVA) with Tukey’s post hoc tests for multiple comparisons or Student’s *t*-test for two-sample comparison was conducted using OriginPro 2018 (v9.5.1) software (OriginLab Corporation, Northampton, MA, USA). Statistical significance was considered at a probability level of 0.05.

## 3. Results and Discussion

**Figure 1:**
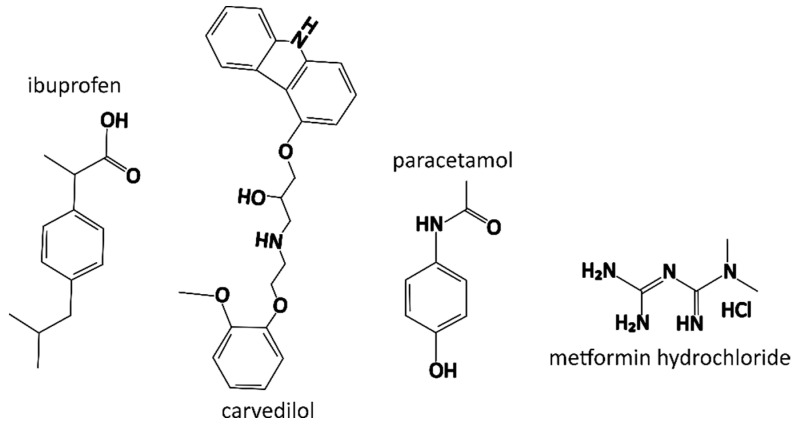
Chemical structures of the model drugs investigated.

In this study, we investigated the influence of a model drug on the most important physicochemical properties of nanofibers. We chose ibuprofen, carvedilol, paracetamol, and metformin hydrochloride (Figure 1), hereafter referred to as metformin, as model drugs that differ in their physicochemical properties (Table 3).

Ibuprofen and carvedilol, despite being practically insoluble in water, demonstrate high intestinal permeability, classifying them as class II drugs according to the Biopharmaceutical Classification System (BCS). Conversely, paracetamol and metformin are both freely soluble in water but exhibit low intestinal permeability, thus being classified as BCS class III drugs [63,64,65,66,67]. However, it is noteworthy that all four investigated drugs exhibit at least slight solubility in ethanol, which is essential for the preparation of ethanol-based solutions for electrospinning. To investigate the impact of the selected model drugs on the nanofiber properties, we adopted the formulation based on PEO and P188 in a 1:1 weight ratio, previously developed by our research group to increase the solubility and dissolution rate of poorly soluble drug lovastatin [54].

### 3.1. Properties of Polymer Solutions

The assessment of the rheological properties of the polymer solutions for electrospinning revealed that the physicochemical properties of the model drug did not significantly affect the rheological behavior of the polymer solution (Figure S1). The dynamic viscosity of the prepared polymer solutions, with or without the model drug, was independent of shear rate and exhibited viscoelastic Newtonian behavior [68,69].

It was observed that carvedilol, paracetamol, and metformin hydrochloride did not significantly alter the dynamic viscosity of the polymer solution compared to the drug-free polymer solution with a total polymer concentration of 3% (*w*/*w*) (formulation 0), which exhibited a dynamic viscosity of ~26.8 mPas (Table 4). On the other hand, the addition of ibuprofen to the polymer solution resulted in a slight decrease in the solution’s dynamic viscosity compared to the drug-free polymer solution (Table 4), possibly due to its pronounced solubility in ethanol. According to Ph. Eur., both ibuprofen and paracetamol are freely soluble in ethanol. However, ibuprofen exhibits approximately seven times greater solubility in ethanol than paracetamol [70,71], making it the most soluble in ethanol among the investigated model drugs.

The dynamic viscosities of the polymer solutions under investigation were consistent with literature data [18,72], confirming their suitability for electrospinning of nanofibers. Regardless of the model drug incorporated, all polymer solutions maintained dynamic viscosities within an optimal range. This is crucial for preventing issues such as poor flow of the polymer solution and the formation of beaded or discontinuous nanofibers, which can occur due to excessively high or low dynamic viscosities [73,74].

The evident prevalence of plastic properties over elastic properties, which is a prerequisite for successful polymer solution spinnability [5], was observed for all polymer solutions regardless of the used model drug (Figure S2). However, the addition of ibuprofen to the polymer solution had a minor impact on the elastic properties of the polymer solution, potentially due to its superior solubility in ethanol compared to the other investigated model drugs [70,71]. In contrast, the addition of carvedilol, paracetamol, or metformin to the polymer solution significantly affected the dependency of the polymer solution’s elastic properties on the angular frequency (Figure S2).

The model drugs, except metformin, had negligible effects on the electrical conductivity of the investigated polymer solutions (Table 4). The elevated electrical conductivity observed in the metformin-containing polymer solution was attributed to the addition of metformin in a salt form. The literature data show that the addition of salts in the polymer solution for electrospinning may enhance the electrical properties and increase the electrical conductivity more than 50 times for pure PEO solutions [75], which is in line with the increased electric conductivity of polymer solution for formulation MET (Table 4).

While existing literature suggests that increased electrical conductivity might result in finer nanofibers with fewer beads and potentially undesired jet instability during electrospinning, resulting in broader distributions of fiber diameters [73,76], our previous research demonstrated that polymer solutions with even higher electrical conductivities remain suitable for nanofiber formation using the electrospinning method [18]. Thus, the increased electrical conductivity of the polymer solution in formulation MET did not raise concerns regarding nanofiber formation. Conversely, the low electrical conductivities of the other investigated solutions might present challenges for the electrospinning process [10].

### 3.2. Morphology of Electrospun Products

**Figure 2:**
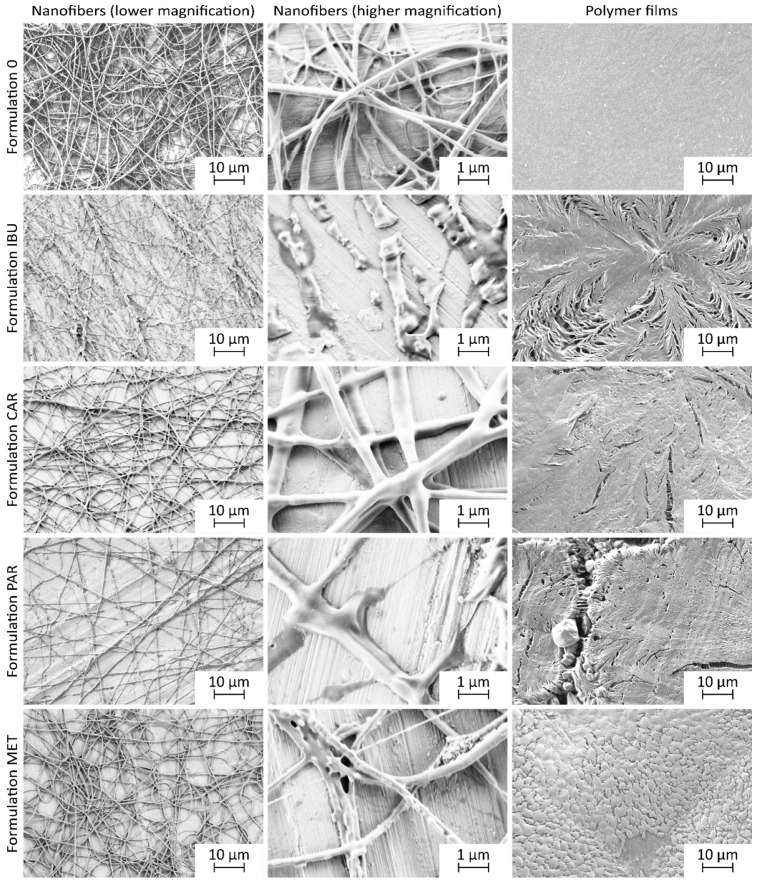
Representative SEM images of the electrospun nanofibers and corresponding polymer films of all investigated formulations.

**Figure 3:**
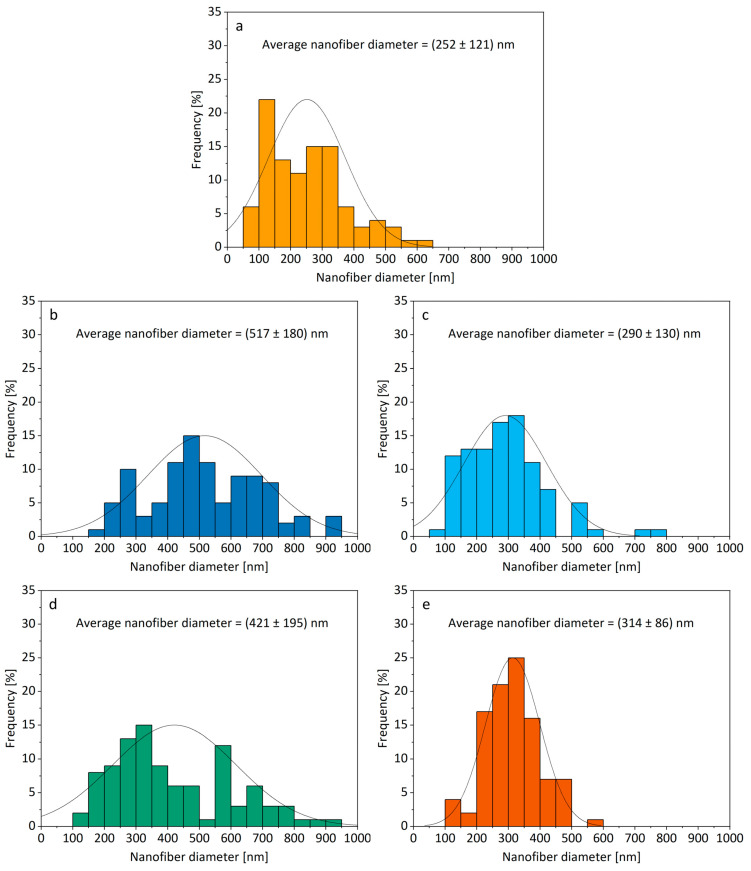
Distribution of nanofiber diameters for (**a**) formulation 0, (**b**) formulation IBU, (**c**) formulation CAR, (**d**) formulation PAR, and (**e**) formulation MET determined based on representative SEM images.

The solubilities of the investigated model drugs in ethanol (Table 3), which was selected as a medium for the preparation of polymer solutions for electrospinning, were shown to affect the rheological properties of the polymer solutions and the morphology of the electrospun products (Figure 2). Thus, the incorporation of metformin hydrochloride or carvedilol, being less soluble in ethanol among the investigated drugs, into nanofibers resulted in the formation of continuous nanofibers with homogeneous nanofiber diameters, resembling the plain nanofibers (formulation 0) (Figure 2 and Figure 3), whereas the incorporation of more ethanol-soluble ibuprofen or paracetamol resulted in the formation of discontinuous or fragmented nanofibers.

The nanofiber morphology in this study differed slightly from our previous findings [72,77]. The nanofibers in the present study were smoother and without beads, which were observed in our previous studies [77]. Since the bead formation is typically associated with the capillary instability of the jet of polymer solution [9], the observed differences in nanofiber morphology may be attributed to the difference in polymer solution composition, namely the use of P188 instead of poloxamer 407, which was used in the previous study. This may have affected the surface tension of the polymer solution, leading to the destabilization of the jet of the viscoelastic polymer solution and subsequent bead formation [78].

Additionally, small particles presumably attributed to metformin were observed on the surface of nanofibers of formulation MET and on the surface of polymer film of the same formulation. A similar observation of small particles on the surface of nanofibers was described in the literature when salt was added to the polymer (PEO) solution for electrospinning [75]. However, according to European Pharmacopoeia, both metformin and carvedilol are slightly soluble in ethanol, with metformin being less soluble than carvedilol [79,80]. The presence of the small particles on the surface of the nanofibers and polymer film of formulation MET could be related to the poor solubility of metformin in ethanol, leading to its precipitation during the formation of nanofibers and polymer film [67]. Despite this, metformin-loaded nanofiber exhibited also the narrowest nanofiber diameter distribution among all investigated formulations (Figure 3). This is in contrast to the literature showing that high electrical conductivity can lead to a broad distribution of nanofiber diameters [81].

The addition of ibuprofen, which had the highest solubility in ethanol among the drugs investigated, into the polymer solution resulted in the formation of fragmented, thick structures spread out on the collector. This was in contrast to the continuous nanofiber formation observed in all other investigated formulations (Figure 2). The literature suggests that the inability to achieve continuous nanofiber formation can be attributed to the low viscosity of the polymer solution [73]. This is in line with our results as the dynamic viscosity of the polymer solution with ibuprofen was significantly lower compared to all other investigated polymer solutions (Table 4). Additionally, the electrical conductivity of the polymer solution with ibuprofen was significantly lower compared to the polymer solutions with carvedilol (formulation CAR) and metformin (formulation MET) (Table 4). The literature suggests that low electrical conductivity could hinder the formation of continuous nanofibers [5]. On the other hand, low electrical conductivity may also lead to the formation of thicker nanofibers [73], as observed in the case of formulation IBU and formulation PAR (Table 4, Figure 3).

Thus, we showed that the physicochemical properties of the incorporated drug, in particular, its solubility in the solvent used to prepare the electrospinning solution, influenced the morphology of the electrospun nanofibers. However, given that the nanofibers were prepared using the same electrospinning conditions, we presume that the differences in the morphology of electrospun nanofibers induced by the incorporation of different drugs might potentially be alleviated by adjustments in process and ambient parameters [5].

### 3.3. Chemical Interactions between Components in Nanofibers

**Figure 4:**
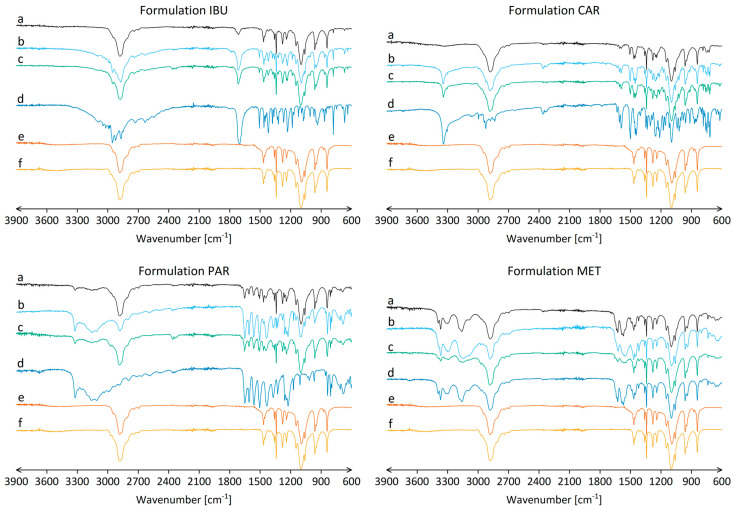
FT-IR spectra in the wavenumber range of 3900–600 cm^−1^ for formulations IBU, CAR, PAR, and MET. (**a**) Nanofibers; (**b**) polymer films; (**c**) physical mixtures of PEO, P188, and selected drug in the weight ratio 2:2:1; (**d**) selected drug; (**e**) polymer PEO; and (**f**) polymer P188.

Chemical interactions between polymers and drugs in the nanofibers were examined, revealing no important changes in characteristic peaks in nanofiber FT-IR spectra of formulations 0 and formulation MET when compared to pure polymers and drugs (Figure 4). However, an absence of the characteristic hydroxyl group stretching vibration above 3000 cm^−1^, observed in the FT-IR spectra of ibuprofen, carvedilol, and paracetamol, was noted in the FT-IR spectra of formulation IBU, CAR, and PAR nanofibers (Figure 4).

The disappearance of the characteristic peak for ibuprofen, carvedilol, and paracetamol in FT-IR spectra of nanofibers of the corresponding formulation suggests the formation of hydrogen bonds between drugs and polymers in the nanofibers [82,83]. Although all the investigated drugs have multiple hydrogen bond donor groups, only ibuprofen, carvedilol, and paracetamol have the hydroxyl hydrogen bond donor group (Figure 1) [63,64,65,66]. Furthermore, PEO is a known hydrogen bond acceptor; thus, it can form hydrogen bonds with hydrogen bond donors (e.g., hydroxyl groups of the investigated drugs) [84]. P188, which has two PEO fragments in its structure, shares this property with PEO [85]. Thus, the change in the FT-IR spectra above 3000 cm^−1^ for ibuprofen, carvedilol, and paracetamol in nanofibers might be associated with the formation of hydrogen bonds with the polymers in the nanofibers. This underscores the significance of the free functional groups present in drugs, particularly the hydroxyl group, when incorporated into nanofibers, as they may significantly contribute to the chemical interactions between drugs and polymers within the nanofibers. The disappearance of the characteristic hydroxyl group stretching vibration above 3000 cm^−1^ was observed only in the FT-IR spectra of nanofibers but not in the FT-IR spectra of polymer films or physical mixtures (Figure 4). This confirms the important role of the electrospinning process in facilitating the formation of hydrogen bonds between drugs and polymers, which is consistent with the literature [86,87]. Similar changes were not observed for metformin, which has no hydroxyl groups but has other hydrogen bond donor groups that show less intense peaks in the FT-IR spectra [88].

Comparison of the FT-IR spectra of pure drugs with those of nanofibers, polymer films, or physical mixtures showed a decrease in the intensity of the characteristic peaks of the drugs (Figure 4). This could be attributed to the relatively high polymer content (80%, *w*/*w*) in the nanofibers compared to the drug loading (20%, *w*/*w*).

### 3.4. Surface Properties of Nanofiber Mats

Incorporation of the hydrophilic drugs, namely paracetamol and metformin, in nanofibers significantly decreased the contact angle of a water droplet on the surface of the nanofiber mat of formulations PAR and MET, compared to the drug-free nanofiber mat of formulation 0 (Table 5). This indicates a significant increase in the hydrophilicity of the nanofiber mats due to the presence of hydrophilic drug in the polymer matrix. Contrarily, the surface properties of nanofiber mats were not significantly affected by the incorporation of more hydrophobic drugs, namely ibuprofen and carvedilol. The contact angle of a water droplet on the surface of the nanofiber mat of formulations IBU and CAR was comparable to that of the drug-free nanofiber mat of formulation 0 (Table 5). Thus, our investigation revealed that the incorporation of hydrophilic drugs into nanofibers importantly affects the surface hydrophilicity of electrospun nanofiber mats (Table 5).

### 3.5. Moisture Content in Nanofibers

**Figure 5:**
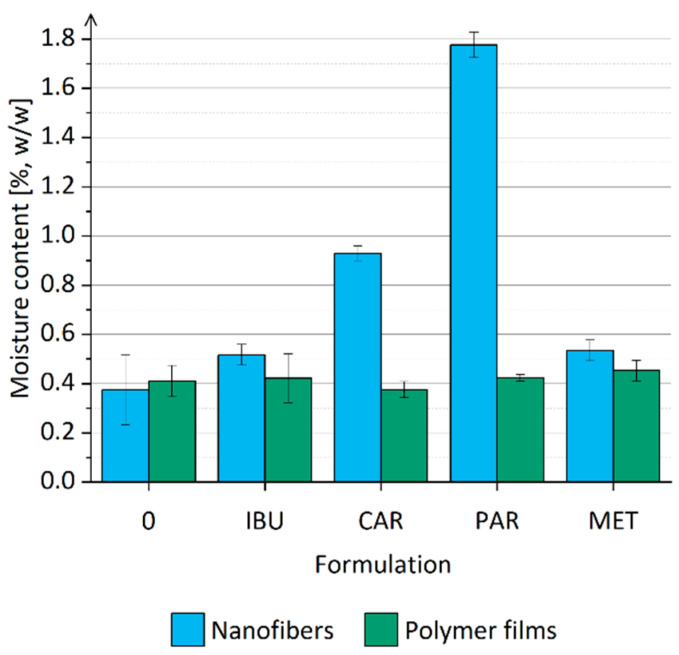
Moisture content in nanofibers and polymer films of formulations 0, IBU, CAR, PAR, and MET after exposure to 46% relative humidity at room temperature for 24 h.

The residual moisture content in all nanofiber and polymer film formulations was below the limit of detection, which is consistent with our previous findings showing that electrospinning of ethanol-based polymer solutions yields a dry electrospun product [18]. Additional experiments revealed that all investigated nanofiber formulations and the corresponding polymer films can sorb moisture when exposed to an environment with 46% relative humidity at room temperature (Figure 5), aligning with our previous studies [18]. The polymer films of all formulations investigated contained ~0.4% (*w*/*w*) moisture after exposure to 46% relative humidity at room temperature for 24 h. In contrast, the moisture content in the nanofibers was dependent on the drug loaded (Figure 5). The incorporation of ibuprofen or metformin did not significantly alter the moisture content compared to the drug-free nanofibers. Significantly higher moisture content in nanofibers after exposure to 46% relative humidity at room temperature for 24 h was observed for nanofibers with carvedilol and paracetamol (Figure 5). Given that paracetamol and carvedilol are known to sorb moisture from the environment [89,90], a characteristic not shared by ibuprofen and metformin [91,92], it can be concluded that the hygroscopic properties of nanofibers are significantly influenced by the hygroscopic nature of the incorporated drug. The significant difference between formulation CAR and formulation PAR in moisture sorption capacity could be attributed to the more pronounced hygroscopic properties of paracetamol compared to carvedilol [89,90]. Since the drugs were incorporated into a matrix of hygroscopic polymers [85] and since the drug-free nanofibers also demonstrated the ability to sorb moisture from the environment, it can be concluded that the hygroscopic behavior of nanofibers is a consequence of the hygroscopicity of the polymers and the drugs. Furthermore, nanofibers exhibited greater hygroscopicity than the polymer films of corresponding formulations, indicating a potential enhancement of formulation hygroscopicity through the formation of a three-dimensional nanofiber mat. However, this was not evident in formulation IBU and formulation MET, which contained less hygroscopic drugs, namely ibuprofen and carvedilol.

### 3.6. Drug Loading in Nanofibers and Drug Entrapment Efficiency

The determined drug loading in all investigated nanofiber formulations was found to be slightly below 20% (*w*/*w*) (Table 6), closely aligning with the theoretical drug loading (Table 1). Thus, the drug entrapment efficiency was slightly below 100% for all formulations (Table 6). Drug loading in our study (20% (*w*/*w*)) was not as high as published in the literature for ibuprofen [62], carvedilol [52,62], or paracetamol [93], but it was significantly higher for metformin compared to the literature data (~17% (*w*/*w*)) [58]. However, the primary aim of our study was not to achieve the maximum drug loading but rather to investigate the effects of drug incorporation on the properties of the nanofibers.

A crucial factor in achieving high entrapment efficiency is preparing the electrospinning polymer solution in a solvent where the drug is soluble, as described in the literature [53,77]. As all the investigated drugs are at least slightly soluble in ethanol (Table 3), which was used as the solvent for the preparation of polymer solutions, our findings align with the literature data. Nonetheless, slightly higher (though not statistically significant) entrapment efficiencies were observed for carvedilol and metformin, which are poorly soluble in ethanol compared to ibuprofen and paracetamol [67]. This suggests that the drug solubility in the solvent used for the preparation of the polymer solution may indeed play a role in achieving high drug entrapment efficiency.

### 3.7. Drug Release from Nanofibers In Vitro

In vitro drug release studies were performed in phosphate buffer (pH 7.4) with 0.1% (*w*/*v*) Tween^®^ 80, which ensured the sink conditions at the neutral pH, in which the different solubility of the drugs did not hinder the drug release. The pH value of 7.4 represents a physiological pH for parenteral and ocular application, which are two of the various possibilities for the application of nanofibers. However, the aim of the drug release studies was not to predict the drug release in vivo but rather to compare the drug release of different drugs from the hydrophilic nanofibers.

**Figure 6:**
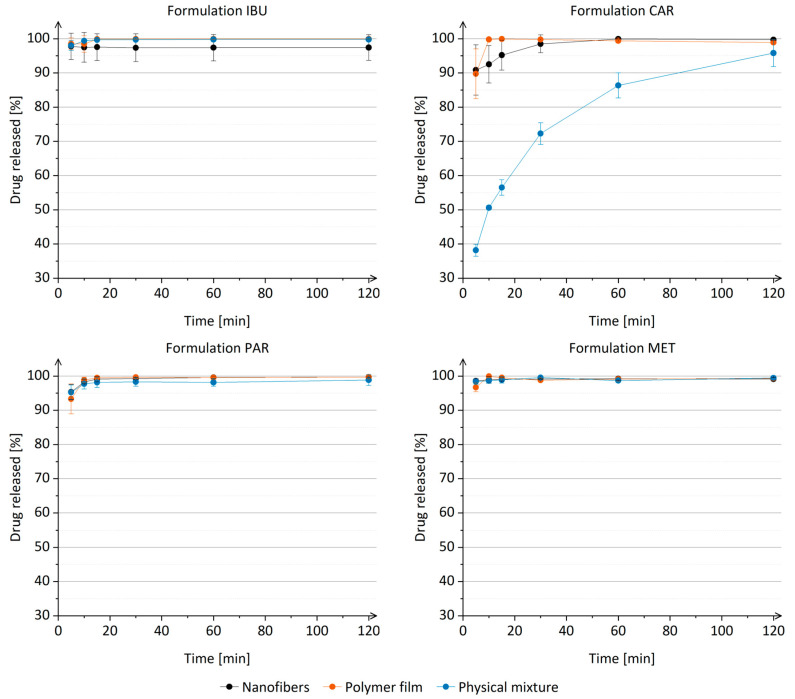
Drug release from nanofibers, polymer films, and physical mixtures of formulation IBU, formulation CAR, formulation PAR, and formulation MET.

In vitro drug release studies revealed that the physicochemical properties of the drugs do not affect the drug release from nanofibers (Figure 6). Similar observations also applied to the reference formulations represented by the polymer films (Figure 6). This highlights the potential of the formulation, based on the combination of hydrophilic polymers (i.e., PEO and P188), as an effective drug delivery system capable of facilitating immediate drug release for all drugs investigated, as nearly 100% of the drug was released from all the nanofiber formulations in 15 min (Figure 6). The rapid drug release observed across all formulations can be attributed to the inherent properties of the hydrophilic polymers employed. Specifically, PEO is recognized for its ability to enable the rapid dissolution of nanofibers, leading to accelerated drug release [94,95]. Additionally, the surface-active properties of P188 play a role in facilitating nanofiber dissolution and subsequent rapid drug release [85].

Moreover, it was shown that the drug release from nanofibers might be linked to the dispersibility of nanofibers, which is affected by the incorporated drug. However, no clear correlation could be established between the physicochemical properties of the drug and the dispersibility of nanofibers. Nanofibers of formulations 0, IBU, and MET were dispersed faster in phosphate buffer (pH 7.4) with 0.1% (*w*/*v*) Tween^®^ 80 than nanofibers of formulations CAR and PAR. A similar, though not statistically significant, relationship was also observed for drug release from nanofibers (Figure 6). However, the dispersibility and in vitro drug release studies were performed in phosphate buffer (pH 7.4) with 0.1% (*w*/*v*) Tween^®^ 80 to ensure the sink conditions.

The incorporation of carvedilol into nanofibers or polymer film resulted in significantly faster drug release compared to the drug dissolution from the physical mixture of carvedilol (Figure 6), whereas such differences were not observed for nanofibers of formulation IBU, PAR, or MET. It should be pointed out that carvedilol was the only drug among those investigated whose solubility in water and phosphate buffer (pH 7.4) with 0.1% (*w*/*v*) Tween^®^ 80 was significantly enhanced by the presence of PEO and P188 at a concentration of 0.08% (*w*/*v*) (Table S1), which corresponds to the concentration of polymers in the drug release studies. However, no significant difference was observed between carvedilol release from nanofibers and polymer films of comparable thickness. This finding contradicts our previously published results, which demonstrated that carvedilol is released more rapidly from nanofibers than from polymer films [77]. The observed difference could be attributed to the lower hydrophobicity of P188 compared to the poloxamer 407, which was used in our previous study [77,85].

The comparison of the obtained drug release profiles with the literature data revealed similar drug release kinetics irrespective of the polymer composition of nanofibers for ibuprofen [37,96,97], carvedilol [52], and paracetamol [93,97,98]. Remarkably, in a previous study, PEO nanofibers exhibited a slower release of carvedilol compared to the nanofibers examined in our study [53]. This finding implies that P188 plays a crucial role in facilitating the rapid dispersion of nanofibers and subsequent drug release.

## 4. Conclusions

In this study, we have demonstrated the impact of the physicochemical properties of the incorporated drugs on the physicochemical properties of hydrophilic nanofibers. Our findings highlight the solubility of the drug in the solvent used for the preparation of the electrospinning polymer solution and the drug’s salt form as crucial properties affecting the dynamic viscosity and electrical conductivity of polymer solutions. These factors, consequently, importantly affect the morphology of the resulting electrospun products. Moreover, we showed that the incorporation of more hydrophilic drugs into nanofibers enhanced the surface hydrophilicity of nanofibers, whereas the incorporation of more hydrophobic drugs exhibited negligible impact on the surface properties of the nanofibers. However, the physicochemical properties of the incorporated drug had minimal influence on the drug loading, drug entrapment efficiency, and rapid drug release, which were mainly governed by the inherent properties of the employed hydrophilic polymers. In conclusion, our findings emphasize the critical importance of considering drug-specific physicochemical properties when designing and optimizing nanofiber-based drug delivery systems.

## Figures and Tables

**Table 1:** Composition of polymer solutions for electrospinning.

Formulation	Drug Name	Drug [mg]	P188 [mg]	PEO [mg]	Ethanol [g]
0	/	/	150	150	10
IBU	ibuprofen	75	150	150	10
CAR	carvedilol	75	150	150	10
PAR	paracetamol	75	150	150	10
MET	metformin hydrochloride	75	150	150	10

**Table 2:** Parameters of HPLC analyses of investigated drugs.

Parameter/Drug	Ibuprofen	Carvedilol	Paracetamol	Metformin Hydrochloride
Injection volume	20 µL	20 µL	2 µL	1 µL
Mobile phase flow rate	1 mL/min	1 mL/min	1 mL/min	1 mL/min
Chromatographic column	Luna^®^ C18	BetaBasic^®^ C8	Kinetex^®^ C18	Synergi^TM^ Hydro-RP
Column temperature	25 °C	35 °C	40 °C	30 °C
Detection wavelength	222 nm	241 nm	243 nm	237 nm

**Table 3:** Physicochemical properties of the investigated model drugs [63,64,65,66,67].

Property/Drug	Ibuprofen	Carvedilol	Paracetamol	Metformin Hydrochloride
Molecular weight	206.3 g/mol	406.5 g/mol	151.2 g/mol	165.6 g/mol
Appearance	white crystal powder	white crystal powder	white crystal powder	white crystal powder
Water solubility *	practically insoluble	practically insoluble	sparingly soluble	freely soluble
Ethanol solubility *	freely soluble	slightly soluble	freely soluble	slightly soluble
BCS class	II	II	III	III
pKa	4.5	14.0 (acid), 8.7 (base)	9.5 (acid), −4.4 (base)	12.40
logP (experimental)	3.7	3.8	0.5	−2.6

* The solubilities of model drugs in water and ethanol are given according to the Ph. Eur. 11th Ed. (based on the approximate volume of solvent required to dissolve a gram of the drug) [67].

**Table 4:** Dynamic viscosities and electrical conductivities of the investigated polymer solutions at 25 °C.

Formulation	Dynamic Viscosity [mPas]	Electrical Conductivity [μS/cm]
0	26.8 ± 0.6	2.45 ± 0.43
IBU	25.0 ± 0.2	1.53 ± 0.02
CAR	27.0 ± 0.2	2.66 ± 0.23
PAR	27.0 ± 0.2	1.66 ± 0.04
MET	26.1 ± 0.2	687.10 ± 12.60

**Table 5:** The contact angle of a water droplet on the surface of nanofiber mats.

Formulation	Contact Angle [°]
0	53.8 ± 8.8
IBU	51.8 ± 8.0
CAR	50.8 ± 10.9
PAR	29.8 ± 6.4
MET	29.1 ± 8.4

**Table 6:** Drug loading and drug entrapment efficiency for all investigated nanofiber formulations.

Formulation	Drug Loading [%, *w*/*w*]	Entrapment Efficiency [%]
IBU	19.0 ± 0.5	94.8 ± 2.7
CAR	19.5 ± 0.9	97.5 ± 4.3
PAR	18.7 ± 0.1	93.4 ± 0.5
MET	19.9 ± 1.1	99.4 ± 5.5

## Data Availability

The original contributions presented in the study are included in the article/supplementary material, further inquiries can be directed to the corresponding author/s.

## Supplementary Materials

The following supporting information can be downloaded at: https://www.mdpi.com/article/10.3390/nano14110949/s1, Figure S1: (a) Viscosity and (b) flow curves of polymer solutions; Figure S2: Plastic (G″) and elastic (G′) modulus of polymer solutions of (a) formulation 0, (b) formulation IBU, (c) formulation CAR, (d) formulation PAR, and (e) formulation MET; Table S1: Drug solubility in different media; Figure S3: FT-IR spectra in range of 3900–600 cm^−1^, regarding formulation 0, for (a) polymer P188, (b) polymer PEO, (c) physical mixture of PEO, P188, and selected drug (in the weight ratio 2:2:1), (d) polymer film, and (e) nanofibers.
