# Supplementary material for: The Incorporated Drug Affects the Properties of Hydrophilic Nanofibers
Source: Nanomaterials (Basel). 2024 May 28;14(11):949. doi: 10.3390/nano14110949 (PMC11173976; doi:10.3390/nano14110949)
Supplement: Supplementary file 1 [file nanomaterials-14-00949-s001.zip › nanomaterials-3013515-supplementary.pdf]

nanomaterials

*Supplementary materials*

# The Incorporated Drug Affects the Properties of Hydrophilic Nanofibers

**Črt Dragar [1], Robert Roškar [2] and Petra Kocbek [1,*]**

[1] Department of Pharmaceutical Technology, Faculty of Pharmacy, University of Ljubljana, SI-1000 Ljubljana, Slovenia; crt.dragar@ffa.uni-lj.si (Č.D.)
[2] Department of Biopharmaceutics and Pharmacokinetics, Faculty of Pharmacy, University of Ljubljana, SI-1000 Ljubljana, Slovenia; robert.roskar@ffa.uni-lj.si (R.R.)
* Correspondence: petra.kocbek@ffa.uni-lj.si

## S1. Rheological Properties of Polymer Solutions

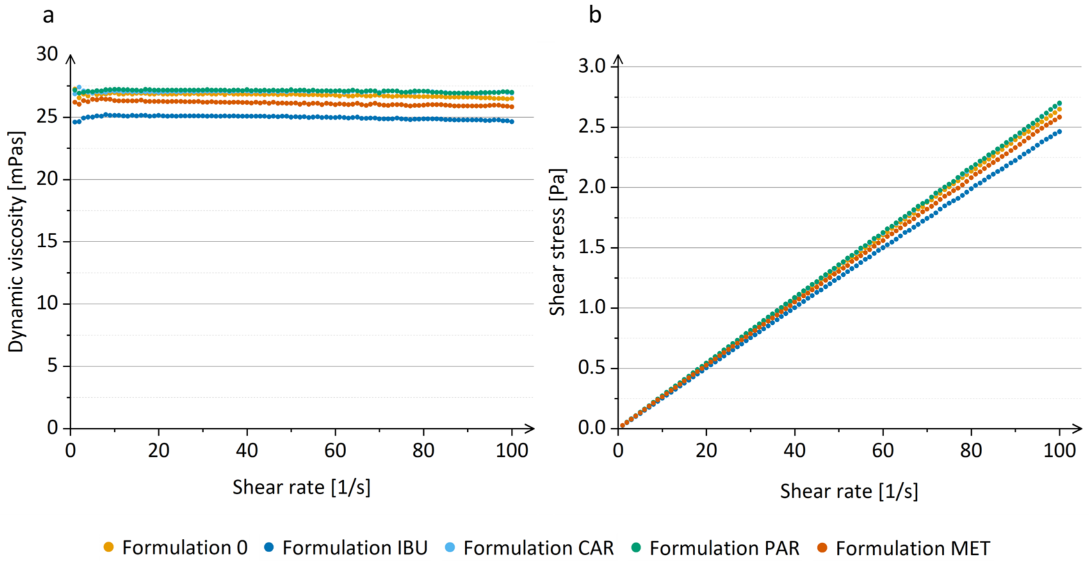


**Figure S1.** (**a**) Viscosity and (**b**) flow curves of polymer solutions.

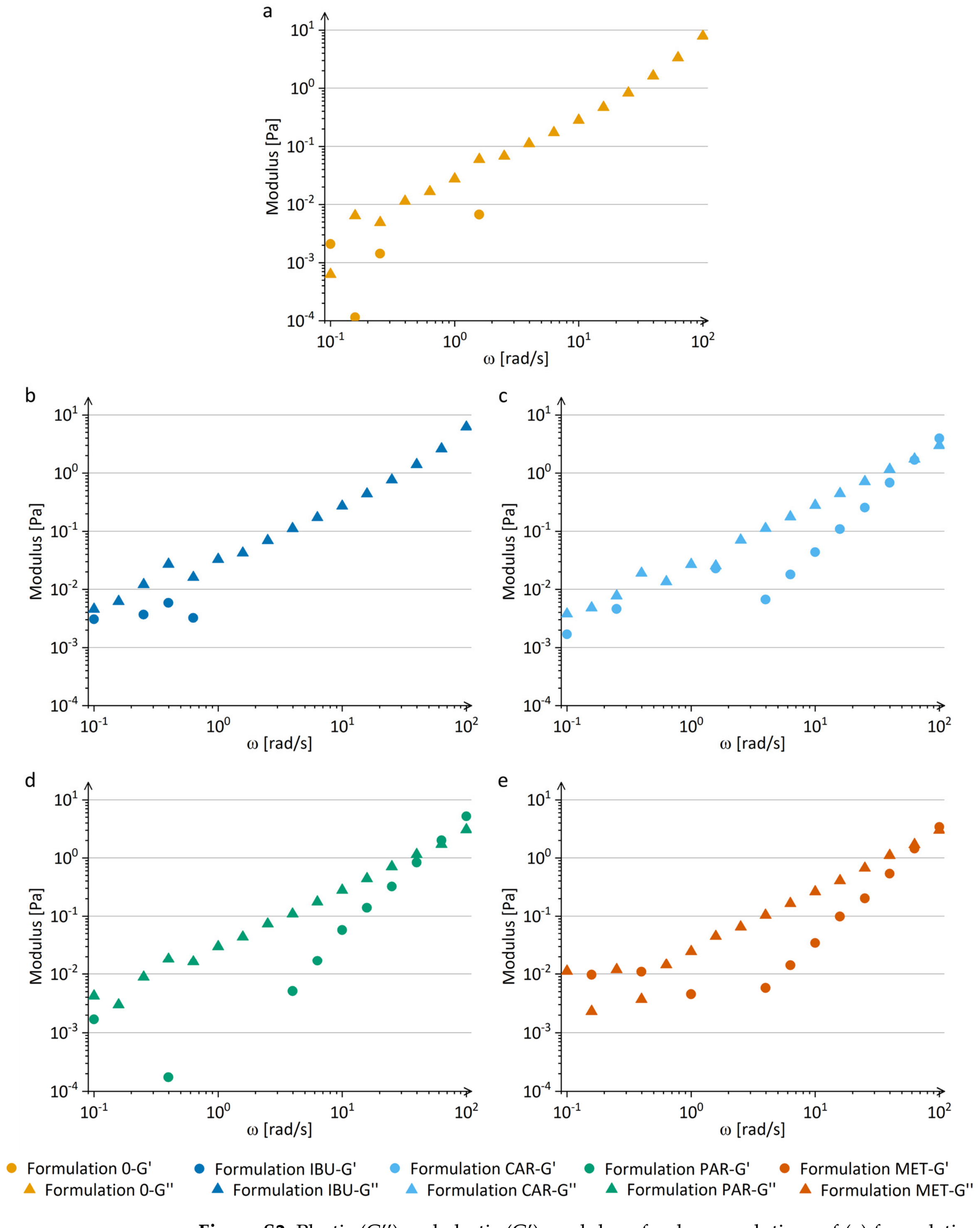


**Figure S2.** Plastic (G′′) and elastic (G′) modulus of polymer solutions of (**a**) formulation 0, (**b**) formulation IBU, (**c**) formulation CAR, (**d**) formulation PAR, and (**e**) formulation MET.

## S2. Evaluation of the Solubility of Drugs

The solubility of the used drug (i.e., ibuprofen, carvedilol, paracetamol, and metformin hydrochloride) was evaluated in water or phosphate buffer (pH 7.4) with 0.1 % (w/v) Tween® 80 with or without dissolved P188 and PEO in the same proportions as present in nanofibers (Table S1). Polymers, PEO and P188 in a weight ratio of 1:1, were dissolved using magnetic stirring at room temperature, and the excess of the investigated drug was

added to the media in a 50 mL Erlenmeyer flask. The samples were shaken at 220 rpm and 37 °C. After 24 h, 48 h, and 72 h, 5 mL of the sample was withdrawn using a 10 mL syringe. The sample was filtered through a 0.20 µm hydrophilic cellulose filter (Minisart® RC, Sartorius, Göttingen, Germany) and diluted with phosphate buffer (pH 7.4) with 0.1 % (w/v) Tween® 80. The drug concentrations were determined with HPLC analysis as described in Section 2.7. The experiments were conducted in triplicates, and the solubility was calculated as the average concentration of the drug at time points in the equilibrium state. The results are given as the average solubility with corresponding standard deviation.

**Table S1.** Drug solubility in different media.

| Drug | Medium | Medium [mL] | Tween® 80 [%, w/v] | PEO [%, w/v] | P188 [%, w/v] | Solubility [mg/mL] |
|---|---|---|---|---|---|---|
| ibuprofen | purified water | 50 | 0.1 | / | / | 0.085 ± 0.002 |
| | phosphate buffer (pH 7.4) | 50 | 0.1 | / | / | 6.711 ± 0.151 |
| | purified water | 50 | 0.1 | 0.04 | 0.04 | 0.100 ± 0.005 |
| | phosphate buffer (pH 7.4) | 50 | 0.1 | 0.04 | 0.04 | 6.691 ± 0.216 |
| carvedilol | purified water | 50 | 0.1 | / | / | 0.013 ± 0.001 |
| | phosphate buffer (pH 7.4) | 50 | 0.1 | / | / | 0.042 ± 0.001 |
| | purified water | 50 | 0.1 | 0.04 | 0.04 | 0.010 ± 0.001 |
| | phosphate buffer (pH 7.4) | 50 | 0.1 | 0.04 | 0.04 | 0.045 ± 0.004 |
| paracetamol | purified water | 50 | 0.1 | / | / | 19.034 ± 0.308 |
| | phosphate buffer (pH 7.4) | 50 | 0.1 | / | / | 19.169 ± 0.894 |
| | purified water | 50 | 0.1 | 0.04 | 0.04 | 20.318 ± 0.466 |
| | phosphate buffer (pH 7.4) | 50 | 0.1 | 0.04 | 0.04 | 19.564 ± 0.808 |
| metformin hydrochloride | purified water | 50 | 0.1 | / | / | 308.665 ± 22.641 |
| | phosphate buffer (pH 7.4) | 50 | 0.1 | / | / | 452.096 ± 26.049 |
| | purified water | 50 | 0.1 | 0.04 | 0.04 | 356.793 ± 41.759 |
| | phosphate buffer (pH 7.4) | 50 | 0.1 | 0.04 | 0.04 | 440.981 ± 37.425 |

## S3. Chemical interactions

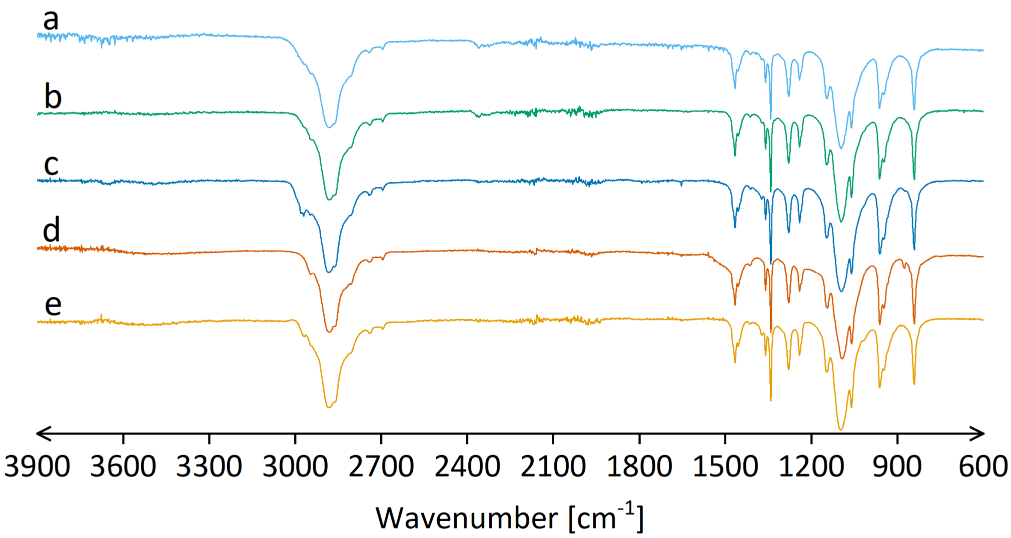


**Figure S3.** FT-IR spectra in range of 3900 - 600 cm$^{-1}$, regarding formulation 0, for (**a**) polymer P188; (**b**) polymer PEO; (**c**) physical mixture of PEO, P188, and selected drug (in the weight ratio 2:2:1); (**d**) polymer film; and (**e**) nanofibers.
